# Bladder-sparing management for high grade noninvasive urothelial carcinoma of the prostate

**DOI:** 10.1016/j.urolonc.2025.04.007

**Published:** 2025-05-24

**Authors:** Alexander C. Martin, Ian M. McElree, Sarah L. Mott, Helen Y. Hougen, Ryan L. Steinberg, Michael A. O’Donnell, Vignesh T. Packiam

**Affiliations:** a Department of Urology, University of Iowa, Iowa City, IA; b University of Iowa Carver College of Medicine, Iowa City, IA; c Holden Comprehensive Cancer Center, University of Iowa, Iowa City, IA; d Department of Surgical Oncology, Rutgers Cancer Institute of New Jersey, New Brunswick, NJ

**Keywords:** Bladder cancer, Prostatic urethral, Gemcitabine, Docetaxel, BCG

## Abstract

**Objectives:**

To characterize the oncologic outcomes in patients with high-grade noninvasive urothelial carcinoma of the prostate (NMIUC-P) treated with intravesical therapy and assess for clinicopathologic features associated with response.

**Subjects and Methods:**

Patients with high-grade NMIUC-P treated with intravesical Bacillus Calmette-Guerin (BCG) or chemotherapy between 2005 and 2021 were retrospectively analyzed. Survival probabilities were estimated using the Kaplan-Meier method. Cox regression was used to evaluate the effect of clinicopathologic and treatment characteristics on high-grade recurrence-free survival (HG-RFS) and progression-free survival (PFS).

**Results:**

A total of 62 patients with median follow-up of 38 months (IQR 19–74) were included. NMIUC-P pathology was carcinoma *in situ* containing in 52 (84%), high-grade Ta in 9 (14%), and high-grade T1 in 1 (2%). Fifty (80%) patients had concomitant bladder UC. Induction regimens were BCG (44%), gemcitabine/docetaxel (42%), and other chemotherapies (14%). HG-RFS was 45%, 43%, and 38% at 1, 2, and 3 years, respectively. Seventeen patients (27%) underwent cystectomy at a median of 12 months, of whom 5 (29%) had ≥T2 and 3 (18%) had *N*+ disease. Among all patients, PFS was 87%, 69%, and 69% at 1, 2, and 3 years, respectively. Cystectomy-free, cancer-specific, and overall survival were 65%, 92%, and 83% at 3 years, respectively. No clinicopathologic or treatment characteristics were significantly associated with HG-RFS.

**Conclusion:**

In a high-risk cohort of patients with NMIUC-P, a select number of patients were able to avoid cystectomy and remain recurrence-free at 3-years after pursuing bladder-sparing intravesical treatment. However, given the high incidence of disease progression, careful patient selection is critical. Further prospective studies are needed to identify markers of response.

## 1. Introduction

Approximately 83,190 new cases of bladder cancer are predicted in 2024 of which 63,070 (76%) are men [1]. Among patients with nonmuscle invasive urothelial carcinoma (UC) of the bladder, prostatic urethral involvement ranges between 16% and 39% across the published literature [2]. Management and outcomes are largely dependent on the depth of invasion within the prostate. Radical cystectomy is recommended for disease invading the prostatic stroma. However, there is no clear consensus on the optimal management of NMIUC-P. Small retrospective studies have demonstrated that bladder-sparing management with intravesical therapy can result in acceptable safety and efficacy [3]. Transurethral resection of the prostate (TURP) is sometimes utilized to ensure adequate staging, eradicate papillary disease, and potentially improve access of antineoplastic agents to deeper submucosal tissues [4,5]. With increasing availability of first-line and salvage intravesical therapies, there is a growing potential for recurrence in the prostatic urethra over time and hence, data supporting management options in this setting are needed [6,7].

Our institution has longstanding experience in bladder-sparing protocols and has utilized selective TURP and intravesical therapy for the treatment of NMIUC-P [8–10]. Herein, we aim to characterize oncologic outcomes in patients with high-grade (HG) NMIUC-P treated with intravesical therapy at our institute between 2005 and 2021 and retrospectively evaluate whether clinicopathologic and treatment characteristics are associated with response.

## 2. Materials and methods

### 2.1. Study design and population

After obtaining the institutional review board approval (#201404766), we retrospectively reviewed all patients with pathologically confirmed HG NMIUC-P at University of Iowa between 2005 and 2021. Patients were eligible if they elected for initial bladder-sparing management after NMIUC-P diagnosis via TURP or prostatic urethral biopsy. Following positive prostatic urethral biopsy, TURP was performed for diagnostic and therapeutic purposes per surgeon discretion. Patients were excluded if they did not intend to undergo at least 5 of 6 total intravesical induction instillations (*n* = 2), did not follow up after induction (*n* = 2), had incomplete records from outside hospital diagnosis (*n* = 2), or underwent upfront radical cystectomy (*n* = 8). Of those undergoing upfront radical cystectomy, 5/8 presented with ≥T2 disease of the prostate and 6/8 had concurrent bladder disease. Two patients were lost to follow-up immediately following cystectomy. Of those with continued surveillance, 3/6 experienced disease metastasis and subsequent bladder cancer-related death.

### 2.2. Intravesical induction therapy

#### 2.2.1. BCG therapy

The BCG treatment was performed as previously described [13]. In brief, patients received intravesical instillation of 1 vial of TICE strain (Organon Teknika Corporation) with or without 50 million units of interferon *α*–2b (IFN-*α*–2b) for approximately 90 to 120 minutes [13]. The BCG dose of the induction cycle was changed to one-third of the dose for some patients during times of limited BCG supply at the institution or when patients experienced substantial side effects.

#### 2.2.2. Chemotherapy

Intravesical therapy protocols for sequential gemcitabine/docetaxel (Gem/Doce) were performed as previously described [11]. In brief, patients received 1 g of gemcitabine in 50 mL sterile water (or normal saline) instilled into the bladder via foley catheter for 90 minutes followed by 37.5 mg of docetaxel in 50 mL normal saline for 90 to 120 minutes [11]. Intravesical induction regimens for all agents were carried out once every week for 6 weeks. If disease-free at first surveillance, monthly maintenance therapy was initiated for up to 24 months and replicated the procedures and dosages used during induction. Other intravesical agents included sequential valrubicin/docetaxel, gemcitabine/cabazitaxel, and single-agent docetaxel were used as described previously [10,12].

#### 2.2.3. Surveillance

The first surveillance was performed 4 to 8 weeks after completion of induction and consisted of either an operative restaging procedure or an office cystoscopy. Operative restaging procedures included a blue light cystoscopy, bladder barbotage washes for cytological testing, FISH analysis, targeted bladder biopsy, random bladder biopsy, and prostatic urethral biopsy in the grooves next to the verumontanum. Bilateral upper tract washes for cytology and retrograde pyelograms were also performed. Patients with negative cystoscopy and suspicious or positive cytology results underwent additional restaging procedures. If the first surveillance was negative for recurrent disease, then the chemotherapy induction patients were considered for monthly maintenance with intravesical treatments for 24 months or until disease recurrence. Patients who had undergone BCG induction received 3 weekly BCG treatments at 3, 9, and 15 months post induction as previously described [13]. When patients remained disease-free, cystoscopy and bladder wash cytological testing was performed every 3 months through 24 months, every 6 months for 24 more months, then annually thereafter. Upper tract imaging was obtained routinely per NCCN guidelines every 12 to 24 months [14]. Cross-sectional imaging by CT or MRI was done annually through year 3, then for cause.

### 2.3. Statistical analysis

Survival probabilities were estimated and plotted using the Kaplan-Meier method. High-grade RFS (HG-RFS) were defined as the time from treatment initiation to HG recurrence. Recurrence was defined as any pathological tumor diagnosis in the prostate or bladder. Cystectomy-free survival (CFS) was defined as the time from treatment initiation to cystectomy. Progression-free survival (PFS) was defined as the time from treatment initiation to muscle-invasive urothelial carcinoma, prostatic stromal invasion, nodal or distant metastasis, or death due to bladder cancer. Patients without recurrence, cystectomy or progression were censored at the last disease evaluation for the respective endpoint. Cancer-specific survival (CSS) and overall survival (OS) were defined as time from treatment initiation to death due to cancer or any cause, respectively. Cox regression model was used to evaluate the effect of patient, disease, and treatment characteristics on HG-RFS and PFS. Statistical analysis was performed using SAS v9.4 (SAS Institute, Cary, NC).

## 3. Results

### 3.1. Clinicopathologic characteristics

A total of 62 patients with a median age of 73 years (IQR 67-78) were included in the analysis. (Table 1) Twenty-three (37%) of diagnoses localizing UC to the prostatic urethra were due to visible disease and 16 (26%) were identified by a biopsy performed due to high-grade bladder wash cytology without visible disease. Fourteen (23%) were identified on biopsies from the prostatic verumontanum grooves that was 1 component of the 3-month restaging following induction intravesical therapy, and 9 (14%) during prostatic resection for other indications [11].

**Fig. 1.**
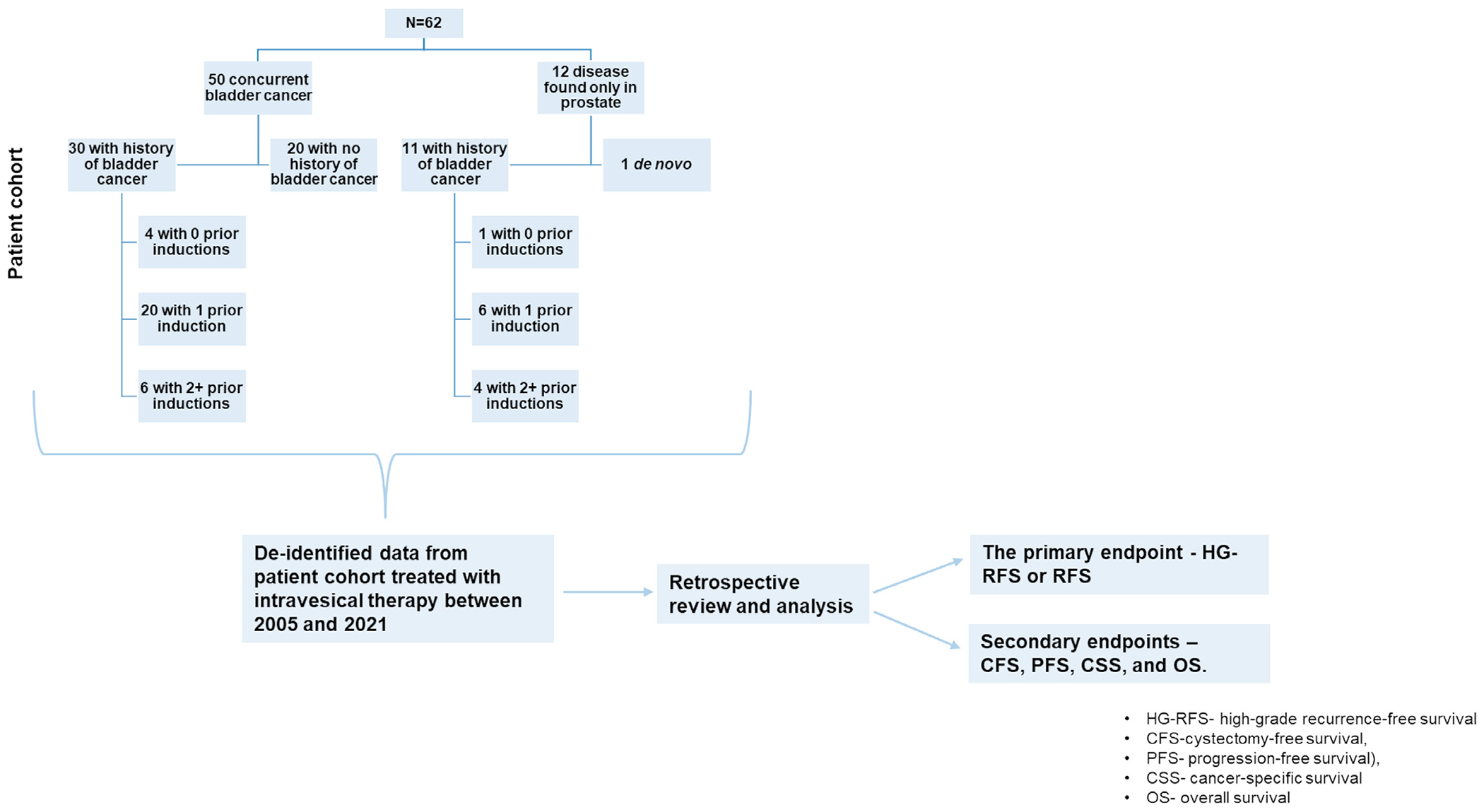
Flow chart of prior disease status and treatment.

At the time of NMIUC-P diagnosis, 50 (81%) patients also had concurrent bladder cancer. Of those patients, 30 had a history of bladder UC, 20 received 1 prior induction intravesical therapy and 6 patients received more than 1 induction course. Twelve (19%) patients had only prostatic UC at the time of diagnosis without any concurrent bladder cancer. Of these patients, 11 had a history of bladder cancer with 10 receiving prior induction intravesical therapy (Fig. 1).

Most of the patients (79%) had pure CIS of the prostatic urethra. The remainder had CIS + Ta (5%), pure TaHG (14%), or pure T1HG (2%) (Table 2). Of patients with concurrent bladder UC, there was wide variation of bladder stage, none of whom had muscle-invasive disease. Notably, 37 patients (74%) had CIS-containing disease. There were 6 (12%) and 7 (14%) patients with pure Ta and T1 HG disease, respectively. Prior to diagnosis of NMIUC-P, 36 (58%) of the entire cohort had undergone intravesical therapy with a median of 1 (IQR 1-2) induction course.

### 3.2. Intravesical treatment of NMIUC-P

Fifty-nine patients completed at least 5 of 6 total induction intravesical treatments. Twenty-seven received a BCG induction course, 26 received a Gem/Doce induction course and 9 received other agents; 5 valrubicin/docetaxel, 3 docetaxel monotherapy, and 1 gemcitabine/cabazitaxel. Five patients received BCG at outside institutions with unknown administration protocols. The median length of time between diagnosis and intravesical treatment was 5 weeks (IQR 4-8). A total of 24 (39%) patients underwent TURP prior to intravesical induction.

### 3.3. Recurrence

**Fig. 2.**
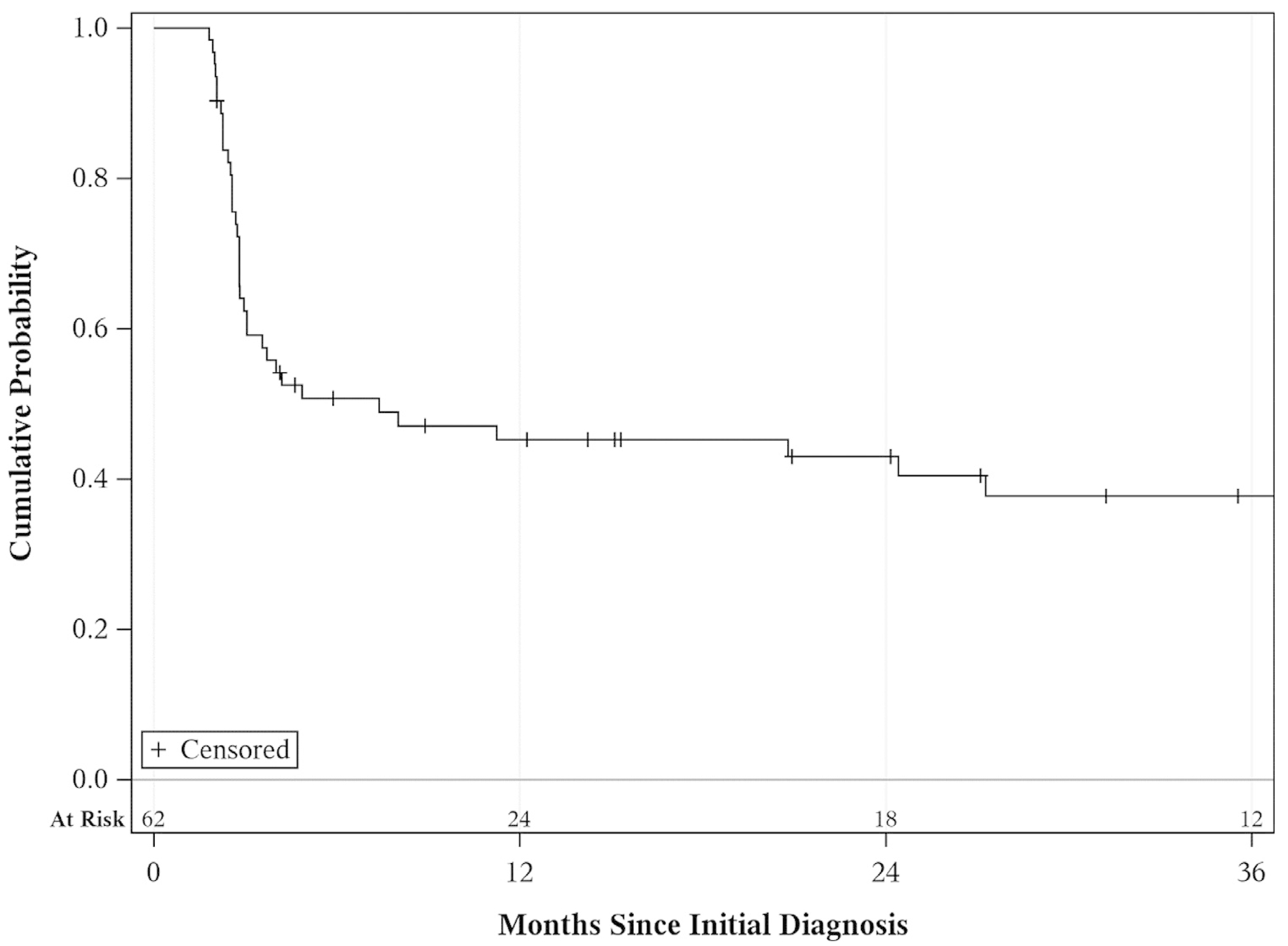
High-grade recurrence-free survival.

**Fig. 3.**
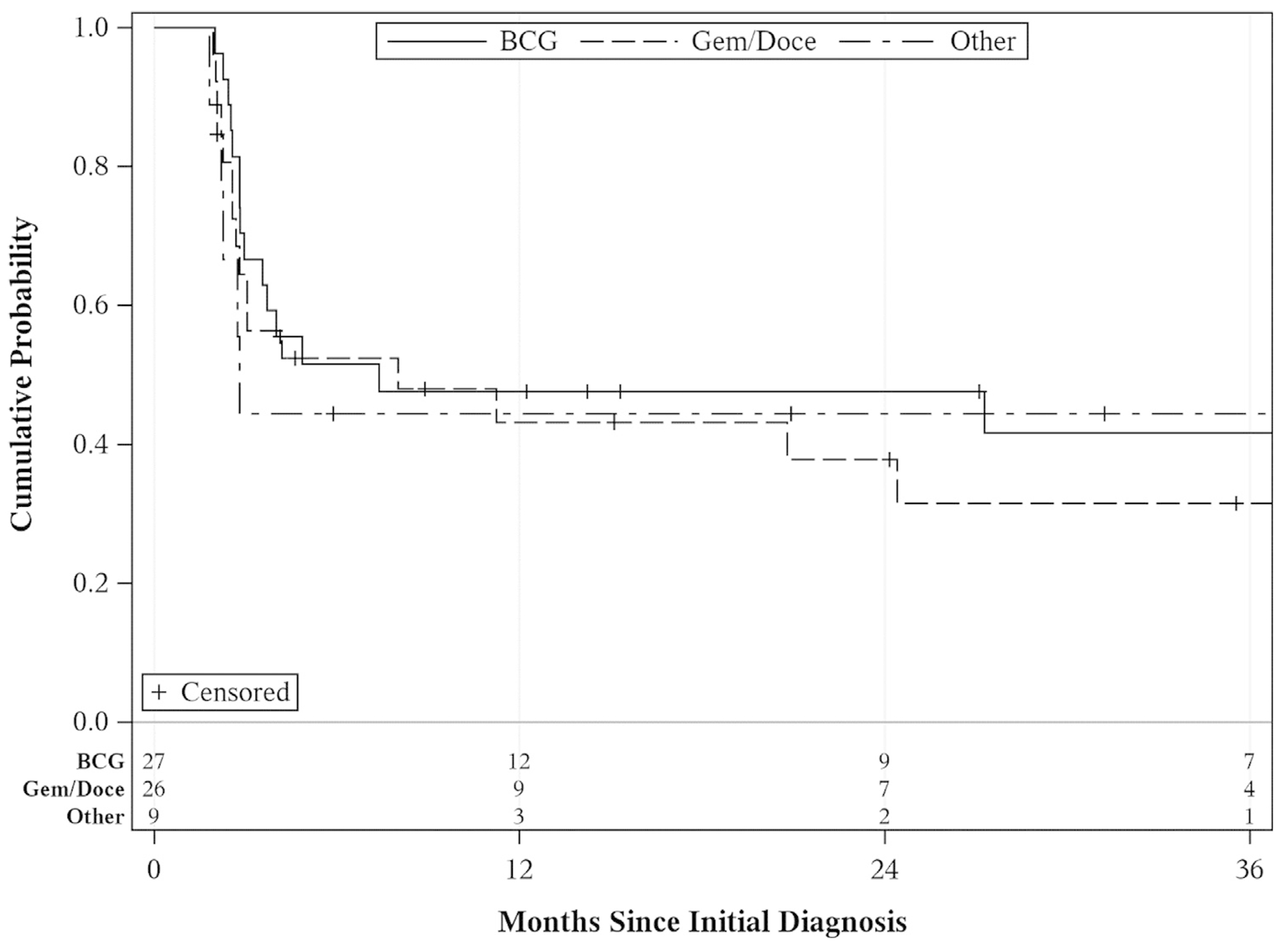
High-grade recurrence free survival stratified by induction therapy.

Thirty-seven patients experienced a HG recurrence. In 15 patients, HG recurrence occurred in the bladder, for 5 patients in the prostate and 17 patients in both bladder and prostate. Overall HG-RFS was 45% at 1 year, 43% at 2 years, and 38% at 3 years (Fig. 2). Prostatic urethral HG-RFS was 67%, 64%, and 58% as compared to bladder HG-RFS at 57%, 51%, and 46% at 1, 2, and 3 years, respectively. There was no statistically significant association between clinicopathologic features including age, BMI, diabetes, smoking status, prior intravesical therapy, prior or current upper tract UC or concurrent bladder disease and HG-RFS (Table 3). In addition, there was no difference in HG-RFS when the cohort was stratified by induction intravesical therapy agent (Fig. 3).

### 3.4. Cystectomy

Of the 62 patients, 17 underwent radical cystectomy at a median of 12 months (IQR 6, 13), 10 due to recurrence, 6 due to progression, and 1 for end-stage bladder symptoms of frequency/urgency. A summary of patients undergoing cystectomy is presented in Supplementary Table 1. CFS at 1, 2, and 3 years was 86%, 65%, and 65%. On final pathology, 5 patients had T2+ disease and 3 patients had positive lymph nodes. There was no urothelial carcinoma identified within 13 prostate specimens and 4 bladder specimens.

### 3.5. Survival outcomes

The median follow-up since NMIUC-P diagnosis was 38 months (IQR 19–74). Nineteen patients had progression events, 9 with development of muscle invasion, and 10 with metastasis. During follow-up, 18 patients died, 8 from bladder cancer. At 1, 2, and 3 years, PFS was 87%, 69%, and 69%, CSS was 94%, 94%, and 92%, and OS was 93%, 85%, and 83%. On univariate analysis, only age (HR: 1.07, 95% CI: 1.00–1.13, *P* = 0.04) was found to be significantly associated with PFS.

## 4. Discussion

NMIUC-P may become more prevalent as bladder-sparing therapies improve [15]. Our study has several key findings. Utilization of a bladder-sparing approach can potentially avoid high-grade disease recurrence for some patients as evidenced by 3-year HG-RFS of 38%. Second, those who remain disease-free after 1 year are more likely to have a durable response. Next, we did not identify any clinicopathologic features associated with HG-RFS. The CFS at 3 years was 65%, and thus, careful patient selection and timely cystectomy in nonresponders is needed.

Prior studies have reported treatment of NMIUC-P managed with bladder-sparing protocols, but their findings have limitations of small size and inconsistent definitions for response [4,5,16–18]. While most reports have utilized BCG, our institution has additionally utilized other intravesical single and combination chemotherapy regimens including gemcitabine, docetaxel, valrubicin, and others [10,12]. A recent meta-analysis of intravesical therapy for NMIUC-P demonstrated a global pooled complete response rate of 60%, although the definitions of complete response and failure to therapy used across the studies were inconsistent [3]. The recurrence rate was high when considering both the bladder and prostate.

Progression is a major concern with bladder-sparing protocols, and this concern is heightened in the case of extravesical prostatic involvement. Our cohort was heavily pretreated compared to most studies with 58% having undergone prior intravesical induction for bladder cancer. However, despite the inclusion of a larger high-risk cohort in our series, oncologic outcomes were comparable to previously published series; an estimated 31% of patients experienced progression at 3 years [3]. Of the 17 patients pursuing radical cystectomy after bladder-sparing therapy, 5 experienced disease progression to T2+ disease, 3 with positive nodes. These findings further highlight the high-risk nature of this cohort and the need for accurate predictors of response which were unable to be elucidated here. Among patients who underwent upfront radical cystectomy, the majority (5/8) had locally advanced disease (≥T2), and among those with continued surveillance, 50% (3/6) developed metastases and ultimately died of bladder cancer. These results align with prior studies indicating that prostatic stromal invasion is a significant adverse prognostic factor.

We did not identify clinicopathologic characteristics or interventions that were associated with response. TURP is recommended by many to accurately stage the extent of prostatic involvement, and importantly may have a therapeutic effect—but we did not find a statistically significant association between TURP status and HG-RFS or PFS. This is consistent with the recent meta-analysis by Kokorovic et al. [3]. Furthermore, some experts recommend tailoring management depending on the presence of prostatic ductal involvement in the disease [14,19]. Our study did not find a statistically significant difference in HG-RFS or PFS whether ductal involvement was present, which is also congruent with prior studies [3]. Ultimately, some associations may not have been found due to limitations in sample size.

As intravesical therapy options expand, there are “sanctuary sites” of UC identified, particularly in the prostatic urethra and upper tract. New upper tract urothelial carcinoma was found in 15% of patients while on surveillance after intravesical BCG for NMIUC-P [20]. Series of promising intravesical therapy regimens have also demonstrated failure in these sites [6].

Many patients express a strong desire to retain their bladder and our data suggests there may be a safe window to attempt bladder-sparing therapy with acceptable response rates. However, it cannot be overly stressed that this approach requires careful patient selection and aggressive management of recurrences to potentially avoid progression. Thirty-one percent of patients ultimately progressed by 3 years, and several patients who underwent cystectomy had nonorgan confined disease. Half of the patients had high-grade recurrence within 6 months of induction. Ten of the 19 total patients with progression developed metastasis. It is important to note, however, that recurrence and progression were co-occurring events and thus time to recurrence cannot be used to predict progression. This may be a hypothesis generating information wherein timely cystectomy in early HG recurrence may prevent adverse progression events, however, a prospective study and/or alternative study design is required to evaluate such a relationship.

Regarding patient selection, our cohort was particularly high-risk. Notably, 79% of the cohort presented with pure CIS—a pan-urothelial disease associated with a heightened risk of recurrence and progression. Indeed, many patients reported here had a history of or concurrent bladder and upper tract urothelial carcinoma. These factors complicate the ability to achieve a complete response and contribute to the challenges of bladder-sparing management. We observed that for patients who experienced recurrence, it mostly occurred within the first 6 to 12 months following intravesical therapy. After 12 months, those who are disease-free have a higher chance of durable response. The findings highlight the importance of meticulous early surveillance, particularly for patients with extensive CIS involvement and multiple prior recurrences.

This study has certain limitations, including its retrospective nature at a single institution and heterogeneity of intravesical treatments. Firstly, our results are based off the data from a high-volume institute and referral center for bladder-sparing therapy for NMIBC with rigorous surveillance protocols and hence might have restricted replicability and application at low-volume NMIBC centers. Secondly, the retrospective nature of this study from a single institute may have introduced a selection bias and confounding variables. Though there were no significant differences in survival outcomes when comparing the induction intravesical agents used, there was heterogeneity amongst the BCG group as some patients received one-third of the initial dose due to supply shortage and others adjunctive interferon alpha or granulocyte-macrophage colony stimulation factor. Additionally, the administration of BCG was not standardized as several patients received BCG administration at outside institutions with unknown administration protocols. The small sample size limits the statistical power and precludes detecting clinically meaningful differences in subgroup analysis. A prospective study is therefore warranted to validate these retrospective results.

## 5. Conclusion

Bladder-sparing treatment with intravesical therapy for NMIUC-P can effectively prevent recurrence in select high-risk patients. However, there is a significant risk of disease progression, and thus careful patient selection and timely cystectomy in nonresponders is paramount. We were not able to identify factors associated with treatment response or disease progression. Prospective evaluation is warranted to further elucidate the use of bladder-sparing therapy for patients with NMIUC-P.

## Supplementary Material

1

Supplementary material associated with this article can be found in the online version at https://doi.org/10.1016/j.urolonc.2025.04.007.

## Figures and Tables

**Table 1:** Clinicopathologic Characteristics of Patients Who Received Intravesical Induction for High-Grade UC of the Prostatic Urethra.

	Median (IQR)	

Age	73 (67, 78)	
	N	(%)
Sex		
Male	62	(100)
Race		
Native American	1	(2)
White	59	(98)
Missing	2	-
Ethnicity		
Non-Hispanic	58	(100)
Missing	4	-
Diabetes		
Yes	24	(39)
No	38	(61)
Smoking status		
Never	20	(32)
Former	35	(57)
Current	7	(11)
Immunodeficiency		
Yes	4	(6)
No	58	(94)
History of bladder cancer		
Yes	41	(66)
No	21	(34)
Prior induction		
Yes	36	(58)
No	26	(42)
Number of prior inductions		
0	26	(42)
1	26	(42)
2+	10	(16)
Preinduction TURP		
Yes	24	(39)
No	38	(61)
Ductal involvement		
Yes	10	(16)
No	52	(84)
Time from bladder cancer diagnosis		
<6 months	27	(44)
6+ months	34	(56)

**Table 2:** Pathology of the Prostatic Urethra and Bladder.

Prostatic Urethral Pathology (*N* = 62)		Concurrent Bladder Cancer (*N* = 50)	
CIS only	49 (79%)	CIS only	20 (40%)
TaHG	9 (14%)	TaHG	6 (12%)
TaHG + CIS	3 (5%)	TaHG + CIS	8 (16%)
T1HG	1 (2%)	T1HG	7 (14%)
T1HG + CIS	0	T1HG + CIS	9 (18%)

**Table 3:** Cox Regression Analysis of Clinicopathologic and Treatment Characteristics Associated With High-Grade Recurrence-Free Survival.

	N	Hazard Ratio	95% CI		*P*-value
Age	62	1.02	0.98	1.05	0.35
BMI	61	1.00	0.95	1.04	0.86
Diabetes					
Yes	24	1.19	0.63	2.23	0.59
No	38	Ref	-	-	
Smoking status					
Former	35	1.88	0.89	3.97	0.25
Current	7	1.56	0.53	4.60	
Never	20	Ref	-	-	
Time to bladder cancer diagnosis					
<6 months	27	0.93	0.49	1.76	0.82
6+ months	34	Ref	-	-	
Prior or current CIS					
Yes	54	0.63	0.28	1.43	0.27
No	8	Ref	-	-	
Prior or current T1HG					
Yes	28	1.07	0.57	2.01	0.83
No	34	Ref	-	-	
Number of prior inductions					
1	26	1.10	0.55	2.19	0.92
2+	10	0.92	0.38	2.23	
0	26	Ref	-	-	
Concurrent bladder cancer					
Yes	50	1.20	0.53	2.72	0.66
No	12	Ref	-	-	
Preinduction TURP					
Yes	24	1.17	0.63	2.19	0.62
No	38	Ref	-	-	
Ductal involvement					
Yes	10	0.95	0.40	2.27	0.91
No	52	Ref	-	-	
Induction regimen					
Gem/Doce	26	1.29	0.67	2.51	0.75
Other	9	1.18	0.43	3.19	
BCG-containing	27	Ref	-	-	

## References

[1] Society AC. Key Statistics for Bladder Cancer. 2023 Available from: https://www.cancer.org/cancer/types/bladder-cancer/about/key-statistics.html 2023. Accessed January 1, 2024.

[2] Amin MB, Greene FL, Edge SB, Compton CC, Gershenwald JE, Brookland RK, The Eighth edition AJCC Cancer Staging Manual: continuing to build a bridge from a population-based to a more “personalized” approach to cancer staging. CA Cancer J Clin 2017;67(2):93–9. 28094848 10.3322/caac.21388

[3] Kokorovic A, Westerman ME, Krause K, Hernandez M, Brooks N, Dinney CPN, Revisiting an old conundrum: a systematic review and meta-analysis of intravesical therapy for treatment of urothelial carcinoma of the prostate. Bladder Cancer 2021;7(2):243–52. 34195319 10.3233/BLC-200404 PMC8204151

[4] Orihuela E, Herr HW, Whitmore WF Jr.. Conservative treatment of superficial transitional cell carcinoma of prostatic urethra with intravesical BCG. Urology 1989;34(5):231–7. 2815442 10.1016/0090-4295(89)90314-2

[5] Bretton PR, Herr HW, Whitmore WF Jr., Badalament RA, Kimmel M, Provet J, Intravesical bacillus Calmette-Guerin therapy for in situ transitional cell carcinoma involving the prostatic urethra. J Urol 1989;141(4):853–6. 2926879 10.1016/s0022-5347(17)41031-7

[6] DeCastro GJ, Sui W, Pak JS, Lee SM, Holder D, Kates MM, A phase I trial of intravesical cabazitaxel, Gemcitabine and Cisplatin for the treatment of nonmuscle invasive bacillus Calmette-Guérin unresponsive or recurrent/relapsing urothelial carcinoma of the bladder. J Urol 2020;204(2):247–53. 32118506 10.1097/JU.0000000000000919 PMC8464350

[7] Packiam VT, Richards J, Schmautz M, Heidenreich A, Boorjian SA. The current landscape of salvage therapies for patients with bacillus Calmette-Guérin unresponsive nonmuscle invasive bladder cancer. Curr Opin Urol 2021;31(3):178–87. 33742981 10.1097/MOU.0000000000000863

[8] Yong C, Mott SL, Steinberg RL, Packiam VT, O’Donnell MA. A longitudinal single center analysis of T1HG bladder cancer: an 18 year experience. Urol Oncol 2022;40(11):491.e1–9. 10.1016/j.urolonc.2022.06.007 35831215

[9] Chevuru PT, McElree IM, Mott SL, Steinberg RL, O’Donnell MA, Packiam VT. Long-term follow-up of sequential intravesical gemcitabine and docetaxel salvage therapy for non-muscle invasive bladder cancer. Urol Oncol 2023;41(3):148.e1–7. 10.1016/j.urolonc.2022.10.030 36456454

[10] McElree IM, Packiam VT, Steinberg RL, Mott SL, Gellhaus PT, Nepple KG, Sequential intravesical valrubicin and docetaxel for the salvage treatment of non-muscle-invasive bladder cancer. J Urol 2022;208(5):969–77. 35830552 10.1097/JU.0000000000002848

[11] McElree IM, Steinberg RL, Mott SL, O’Donnell MA, Packiam VT. Comparison of sequential Intravesical Gemcitabine and Docetaxel vs Bacillus Calmette-Guérin for the treatment of patients with high-risk non-muscle-invasive bladder cancer. JAMA Netw Open 2023;6(2):e230849. 36853609 10.1001/jamanetworkopen.2023.0849 PMC9975907

[12] Packiam VT, McElree I, Steinberg RL, Prescott A, Garje R, Zakharia Y, PD26–07 Sequential endoluminal cabazitaxel and gemcitabine with Pembrolizumab for docetaxel-unresponsive non-muscle invasive urothelial carcinoma of the upper and lower urinary tracts. J Urol 2022;16(14):2561.

[13] Joudi FN, Smith BJ, O’Donnell MA. Final results from a national multicenter phase II trial of combination bacillus Calmette-Guérin plus interferon alpha-2B for reducing recurrence of superficial bladder cancer. Urol Oncol 2006;24(4):344–8. 16818189 10.1016/j.urolonc.2005.11.026

[14] Flaig TW, Spiess PE, Abern M, Agarwal N, Bangs R, Boorjian SA, NCCN Guideline^®^ insights: bladder cancer, version 2.2022. J Natl Compr Canc Netw 2022;20(8):866–78. 35948037 10.6004/jnccn.2022.0041

[15] Mohamed NE, Chaoprang Herrera P, Hudson S, Revenson TA, Lee CT, Quale DZ, Muscle invasive bladder cancer: examining survivor burden and unmet needs. J Urol 2014;191(1):48–53. 23911603 10.1016/j.juro.2013.07.062 PMC4286331

[16] Canda AE, Tuzel E, Mungan MU, Yorukoglu K, Kirkali Z. Conservative management of mucosal prostatic urethral involvement in patients with superficial transitional cell carcinoma of the bladder. Eur Urol 2004;45(4):465–9:discussion 9-70. 15041110 10.1016/j.eururo.2003.12.014

[17] Taylor JH, Davis J, Schellhammer P. Long-term follow-up of intravesical bacillus Calmette-Guérin treatment for superficial transitional-cell carcinoma of the bladder involving the prostatic urethra. Clin Genitourin Cancer 2007;5(6):386–9. 17956711 10.3816/CGC.2007.n.021

[18] Gofrit ON, Pode D, Pizov G, Zorn KC, Katz R, Shapiro A. Prostatic urothelial carcinoma: is transurethral prostatectomy necessary before bacillus Calmette-Guérin immunotherapy? BJU Int 2009;103(7):905–8. 19021623 10.1111/j.1464-410X.2008.08210.x

[19] Babjuk M, Burger M, Capoun O, Cohen D, Compérat EM, Dominguez Escrig JL, European Association of Urology Guidelines on non-muscle-invasive bladder cancer (Ta, T1, and Carcinoma in Situ). Eur Urol 2022;81(1):75–94. 34511303 10.1016/j.eururo.2021.08.010

[20] Celestino F, Verri C, Carlo FD, Zampa G, Pagliarulo V, Masedu F, Urothelial carcinoma of the prostatic urethra: long-term follow-up study. J Clin Oncol 2015;33(15_suppl):e15639.

