# Supplementary material for: Bladder-sparing management for high grade noninvasive urothelial carcinoma of the prostate
Source: Urol Oncol. Author manuscript; Available in PMC 2026 May 28. (PMC13218301; doi:10.1016/j.urolonc.2025.04.007)
Supplement: 1 [file NIHMS2174838-supplement-1.docx]

**Indication and Pathology of Patients who Underwent Radical Cystectomy after Initial Intravesical Induction Therapy**

| **Presenting Pathology** | | **Treatment Group** | **Cystectomy Indication** | **Time to HG Recurrence** | **Time to Progression** | **Time to Cystectomy** | **Cystectomy Pathology** | | |
| --- | --- | --- | --- | --- | --- | --- | --- | --- | --- |
| Prostate | Bladder |  |  | Months | Months |  | TNM | Prostate | Bladder |
| CIS | CIS | BCG | Recurrence in prostate | 3 | 69 | 12 | pTisN0Mx | T0 | CIS |
| CIS | T1HG + CIS | BCG | Progression in prostate | 4 |  | 8 | pTisN0Mx | T0 | CIS |
| CIS | None | BCG | Progression in bladder |  | 13 | 13 | pT3aN0Mx | T0 | T3 |
| CIS | T1HG + CIS | BCG | Recurrence in bladder | 5 |  | 11 | pTisN0Mx | CIS | T0 |
| CIS | T1HG + CIS | BCG | Progression in prostate | 3 | 8 | 12 | pT0N0Mx | T0 | T0 |
| CIS | None | BCG | Recurrence in prostate | 3 |  | 6 | pTisN0Mx | T0 | CIS |
| CIS | T1HG | BCG | Recurrence in bladder | 3 |  | 12 | pT1N0Mx | T0 | T1 + CIS |
| CIS | None | Gem/Doce | Recurrence in bladder | 2 |  | 5 | pT0NxMx | T0 | T0 |
| CIS | TaHG + CIS | Gem/Doce | Recurrence in bladder and prostate | 21 | 132 | 22 | pTisN0Mx | T0 | CIS |
| CIS | T1HG + CIS | Gem/Doce | Metastasis (Imaging) | 3 | 17 | 17 | pT2bN0Mx | T0 | T2 |
| CIS | T1HG | Gem/Doce | Recurrence in prostate | 3 | 13 | 13 | pT2N2Mx | Ta + CIS | T2 |
| CIS | CIS | Gem/Doce | Recurrence in bladder | 2 |  | 4 | pT1N0Mx | T0 | T1 + CIS |
| CIS | CIS | Gem/Doce | Recurrence in prostate | 2 | 23 | 23 | pT4aN0Mx | T4 | T0 |
| CIS | TaHG + CIS | Gem/Doce | Symptomatic |  |  | 2 | pTisN0Mx | T0 | Ta + CIS |
| CIS | None | Gem/Doce | Recurrence in prostate | 4 |  | 9 | pTisN0Mx | T0 | CIS |
| CIS | T1HG | Val/Doce | Progression in bladder | 2 | 2 | 4 | pT3aN1Mx | CIS | T3 |
| CIS | CIS | Val/Doce | Progression in bladder and prostate | 2 | 12 | 14 | pT1N1Mx | T0 | T1 + CIS |
